# Effectiveness of day care in supporting family caregivers of people with dementia: A systematic review

**DOI:** 10.1590/1980-57642018dn13-030003

**Published:** 2019

**Authors:** Virgínia Lúcia Reis Maffioletti, Maria Alice Tourinho Baptista, Raquel Luiza Santos, Valeska Marinho Rodrigues, Marcia Cristina Nascimento Dourado

**Affiliations:** 1 Universidade Federal do Rio de Janeiro Institute of Psychiatry Center for Alzheimer’s Disease RJ Brazil Center for Alzheimer’s Disease, Institute of Psychiatry, Universidade Federal do Rio de Janeiro, RJ, Brazil.

**Keywords:** day care, Alzheimer disease, caregiver, quality of life, psychological adaptation, centro-dia, demência, doença de Alzheimer, cuidador, qualidade de vida, adaptação psicológica

## Abstract

Day care (DC) provides a break in daily care as a support strategy for family caregivers (FC) of people with dementia (PwD).

**Objective:**

to analyze the support strategies used by the DC for FC, their methodological and theoretical models, as well as the respective benefits for FC burden, coping strategies and quality of life.

**Methods:**

a systematic review following the Prisma methodology was performed on PubMed, PsycInfo, Scopus and SciELO electronic databases in August, 2018. The search keywords were “day care”, “dementia” or Alzheimer disease, “caregiver”, “quality of life” and “psychological adaptation”.

**Results:**

twenty-one studies were included. No randomized controlled trials were found. The provision of education, counseling and support, access to information, the professionals' expertise and the quality of their relationship with DC users were highlighted by caregivers. Compared to standard programs centering on PwD, the integrated program focused on PwD and FC activities showed increased feelings of competence and self-confidence of FC to postpone institutionalization. Heterogeneity in the structure and organization of the DC programs, intervention strategies and theoretical basis was observed.

**Conclusion:**

Integrated programs are a promising approach that addresses the needs and demands of PwD and their FC in a multidimensional manner.

## RESUMO

O centro-dia (CD) permite uma pausa no cuidado diário como estratégia de apoio aos cuidadores familiares (CF) das pessoas com demência (PcD).

**Objetivo:**

analisar as estratégias de suporte aos CF oferecidas pelos CD, seus modelos metodológicos e teóricos, bem como os respectivos benefícios sobre sua sobrecarga, estratégias de enfrentamento e qualidade de vida.

**Métodos:**

uma revisão sistemática utilizando a metodologia Prisma foi realizada nos bancos de dados eletrônicos PubMed, PsycInfo, Scopus e SciELO em agosto, 2018. As palavras chave da pesquisa foram “centro dia”, “demência” ou doença de Alzheimer, “cuidador”, “qualidade de vida” e “adaptação psicológica”.

**Resultados:**

vinte e um estudos foram incluídos. Não foram encontrados estudos randomizados. A oferta de educação, aconselhamento e apoio, o acesso a informações sobre a estrutura e o programa do CD, a expertise dos profissionais e a qualidade da relação destes com os usuários foram aspectos ressaltados pelos cuidadores. Comparados aos programas padrão centrados na PcD, estudos descrevendo o programa integrado, focado nas atividades para PcD e CF, mostraram um aumento no sentimento de competência e autoconfiança do CF e adiamento da institucionalização. Observou-se heterogeneidade na estrutura e organização dos programas de CD, nas estratégias de intervenção e nas bases teóricas.

**Conclusão:**

programas integrados são uma abordagem promissora que atende às necessidades e demandas das PcD e seus CF de forma multidimensional.

By 2030, there is set to be an exponential increase in the costs of caring for people with dementia (PwD) worldwide that will burden social and health services, including long-term care systems.^1^^,^^2^ The informal care offered by the family represents a high economic cost, as it compromises the available time of the family caregiver (FC) for formal work, family and social activities.^2^^,^^3^ For families, the financial impact is significant, considering the costs of medical care, treatment, in addition to formal and informal care.^3^ Economic analysis takes into account the cost/benefit of informal care to provide policymakers with information guiding sustainable actions and care plans which support FC.^2^^,^^3^

In the last twenty years, day care (DC) has been studied as an important health technology in the configuration of support strategies for PwD and their FC.^4^^-^9 Proposed as a service model that provides a friendly environment and care for PwD, while the FC have a break that decreases their exposure to primary stressors, the DC seeks to reduce the burden of care^4^^-^9 and improve the quality of life of the dyad. Studies comparing different community-based temporary care strategies suggest that this care model is the most economical one.^10^

The complexity of the situation experienced by FC, in most cases, generates a burden that compromises their physical and psychological health, impacting their availability for care and can induce early institutionalization of PwD.^11^^-^13 Moreover, the FC burden is worsened by their physical limitations, psychological and psychosomatic complaints, emotional aspects such as anger, fear, guilt, depression,^8^^,^^14^^,^^15^ feelings of dissatisfaction with life and loneliness, doubts about their competence and the effectiveness of their coping strategies. The presence of secondary stressors, such as financial difficulties, the conciliation of care with other family and professional demands, social isolation,^5^^,^^6^ and lack of information on the course and prognosis of dementia, about care and management strategies, as well as the need to adapt to the environment, are also factors that may influence caregiver burden.^7^

Beneficial outcomes have been described for FC who participate in DC, such as decreased stress,^14^ depression,^14^ anger,^15^ more positive daily experiences,^16^ and overall improvement in health and well-being.^5^^,^^8^ Tretteteig et al.,^7^ in a recent integrative review, concluded that DC provide a sense of security and relief for FC, reduce burden and increase motivation for care. Vandepitte et al.,^5^ in a review on the effectiveness of temporary care in supporting FC, observed a decrease in the FC burden and behavioral problems of the PwD. However, DC attendance did not delay institutionalization. Fields et al.^8^ suggest that, given the diversity of service settings and the different models of support offered to caregivers, it is difficult to discern the benefits of reducing FC exposure to primary stressors of those associated with the specific interventions, such as psychosocial and psychoeducational interventions, or individual counseling. This lack of standardization of services makes research in this area a heterogeneous field, while also limits the interpretation of results and their replication.^8^^,^^17^ In addition, the caregiver approaches offered by DC,^8^ include psychosocial and psychoeducational interventions, individual counseling and occasional meetings, but most of the studies do not present the theoretical assumptions that underlie this type of technology.^7^^,^^8^

In view of this, the present review aims to analyze the intervention models for FC that make up the settings of the DC, considering their methodological and theoretical models, as well as the respective benefits for FC burden, coping strategies and quality of life.

## METHODS

This systematic review used the methodology suggested by the Preferred Reporting Items for Systematic Reviews and Meta-Analyses (PRISMA)^18^ criteria. The search was performed according to the following PICOS:^19^

**P** caregivers of people with dementia;

**I** day care;

**C** caregivers assisted by outpatient programs, caregivers without interventions, patients and caregivers without interventions;

**O** stress, burden, quality of life, coping skills and postponement of institutionalization;

**S** cross-sectional, longitudinal, randomized, non-randomized and case-control studies.

The literature search was performed on August 2018, using PubMed, SciELO, PsycINFO and Scopus databases. Studies published from 1998 and 2017 were included. The search keywords included the following MeSH terms: “day care” [MeSH} or (care day) or (partial hospitalization) and “dementia” [MeSH] or Alzheimer disease [MeSH] or (Alzheimer's disease) or (Alzheimer type dementia) and “caregiver” [MeSH] or (family caregiver) or (carers) and “quality of life” [MeSH] or (Health related quality of life) and “adaptation, psychological” [MeSH] or (coping behavior) or (coping skills). A manual search was also performed by revising the reference of all papers selected. The inclusion criteria were: 1. cross-sectional or longitudinal studies, 2. randomized or nonrandomized, 3. with or without a control group, 4. studies on day care for people with dementia with caregiver outcomes, 5. reports written in English or Portuguese.

The exclusion criteria were: 1. Participants with other pathologies; 2. Other community or outpatient care settings; 3. Evaluation of specific therapeutics (medications, physical exercise, occupational therapy etc.); 4. Evaluation of instruments; 5. Literature reviews; 6. Other languages; 7. Studies without full text; 8. Outcomes focused on the PWD; 9. Books, dissertations and theses; 10. Public health policies and epidemiological studies.

### Study selection

The articles were evaluated using the quality score metrics proposed by the Mixed Methods Assessment (MMAT),^20^^,^^21^ a tool for the qualitative evaluation of empirical studies that used qualitative, quantitative or mixed methods. The MMAT establishes corresponding criteria for each research method and scores are rated from 1 to 4, considering the description of each stage of method implementation, using descriptors such as *, **, ***, and ****.

Two authors (VLRM and MCND) screened titles and abstracts to identify eligible papers. We excluded all studies that clearly did not meet all inclusion criteria or that met at least one of the exclusion criteria. Subsequently, two authors (VLRM and MATB) independently reviewed the full publications of the remaining papers and held consensus meetings to discuss any disagreement and to reach a consensus about inclusion. When necessary, a third co-author of this paper (RLS) clarified study eligibility.

The articles were reviewed considering the research procedures and the configuration of DC, the program, its objectives, therapeutic proposals and theoretical basis.

## RESULTS

The initial database searches retrieved 587 articles; 82 were found on PsycINFO, 301 on Scopus, 202 were identified on PubMed and 2 on SciELO. All the authors reviewed the selected documents to reach a consensus.

**Figure 1:**
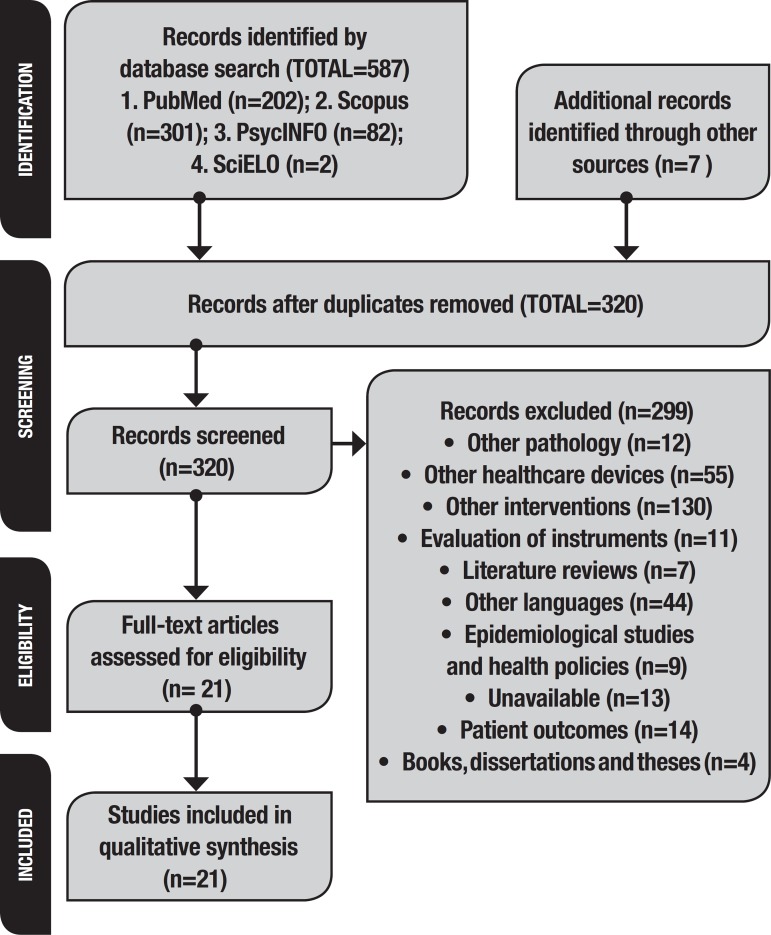
Flow of information through the different phases of the systematic review.

After duplicates were removed (n=274), 299 studies not meeting inclusion criteria were excluded, and fourteen studies were selected for inclusion in this systematic review. Seven further studies were identified by manually searching the references in the selected articles. Twenty-one papers had been selected by the end of this search process (Figure 1). A flow diagram of the study selection process is depicted in Figure 1.

### Methodological aspects

***Place.*** Ten out of the twenty-one studies were conducted in the USA,^22^^-^31 three in the Netherlands,^32^^-^34 one in Norway,^35^ one in Norway and Scotland,^36^ one in Iceland,^37^ one in Hong Kong,^38^ one in Australia,^39^ one in Italy,^40^ one in Sweden^41^ and one in Germany.^42^ No articles on DC in Latin America were found.

***Sample.*** Nineteen studies included FC of both genders, 22^-^24^,^^26^^,^^28^^-^42 predominantly women, while two included females only,^25^^,^^27^ Regarding the type of FC relationship with the PwD, fifteen studies included spouses, children and others,^22^^-^24^,^^26^^,^^28^^,^^31^^-^39^,^^41^ two included only wives and daughters,^25^^,^^27^ whereas four studies did not specify the kinship.^29^^,^^30^^,^^40^^,^^42^ All articles reported that people were diagnosed with dementia, without specifying the causes.

***Study design.*** No randomized controlled trials were found. Six descriptive qualitative studies^23^^,^^24^^,^^32^^,^^35^^,^^36^^,^^38^ and a longitudinal phenomenological interpretative study^37^ were found. Of the six studies, two were longitudinal^37^^,^^38^ and four cross-sectional.^23^^,^^24^^,^^32^^,^^35^ These studies collected reports from caregivers to understand their needs for support and respite, and the role of Day Care in improving the relationship of care, health status and quality of life of the dyad. Ten non-randomized quantitative longitudinal studies with control-group design 22^,^^25^^,^^27^^,^^28^^,^^30^^,^^31^^,^^33^^,^^34^^,^^40^^,^^42^ and four descriptive quantitative studies^26^^,^^29^^,^^38^^,^^41^ were also found. Seven articles evaluated a group of DC user's caregivers comparing them with non-users' family control groups.^22^^,^^25^^,^^27^^,^^30^^,^^31^^,^^40^^,^^42^ Three articles evaluated a group of DC user's caregivers that participated in a special family support program in association with the care provided to PwD via integrated approaches, while control group caregivers participated in the care offered by a standard DC setting which offered activities to PwD only.^28^^,^^33^^,^^34^

***Caregivers' outcomes.*** In the study by de Jong et al.,^32^ the needs and expectations expressed by the FC regarding the assistance offered by the DC emphasized the importance given to the provision of education, counseling and support, access to information about the structure and program of the DC, as well as the professionals' expertise and the quality of their relationship with users. Phillipson et al.^39^ found that the opportunity to learn care strategies through the observation of professionals and the opportunity to engage in other daily and social tasks were valued by caregivers. These aspects contributed to a more positive perception of DC among FC users than non-users.

Seventeen studies observed a reduction in time spent on care activities and in exposure to primary stressors,^22^^-^26^,^^28^^-^31^,^^33^^-^36^,^^38^^-^41 reduced feelings of burden,^22^^,^^25^^,^^30^^,^^31^^,^^33^^,^^40^^,^^41^ worry,^25^^,^^38^^,^^41^ anger,^23^^-^25^,^^31^ affective fluctuation and depression,^22^^-^25^,^^31^ psychological/psychosomatic complaints,^24^^,^^33^^,^^41^ and loneliness.^29^ Increased feelings of competence^28^^,^^34^ and freedom,^35^ strengthening of coping strategies,^34^ improved health status and greater psychological well-being^23^^,^^24^^,^^30^^,^^41^ were also reported. The FC exhibited significantly less distress over behavioral problems of the PwD^26^^,^^40^ and less stress associated with their own non-fulfilment of life expectations.^35^^,^^40^ Having a break increased the positive experiences and free time to stay with other family members or friends,^23^^,^^24^^,^^35^^,^^39^ increased morale^29^ and social activity,^41^ and benefited the relationship between the dyad^36^ and easier cooperation.^35^

Mossello et al.^40^ found no significant between-group difference in depressive symptoms change. Similarly, Zank et al.^42^ found no differences in well-being or burden on standard measures, but, in a semi-structured interview, FC reported substantial positive change promoted by the use of DC.

Kwork et al.^38^ reported that PwD did not improve their capacity for self-care and mobility. Higgins et al.^29^ reported that FC observed no changes in their own quality of life or that of PwD, and reported worsening of cognitive function and behavioral symptoms among PwD.

The observation of improvement in self-esteem and well-being, quality of life and health status, associated with increased social stimulation of the PwD resulted in a secondary benefit for FC health and quality of life.^24^^,^^27^^,^^28^^,^^36^^-^42 FC also reported that participating in the DC promoted a sense of kinship^27^ and maintained the PwD cognitively,^26^^,^^36^^,^^38^^,^^39^ functionally and physically active.^38^ There were also improvements in behavior and sleep changes in the PwD immediately after the use of DC,^26^ and lower use of psychotropic drugs.^40^

A delay in institutionalization was associated with improved health of the dyad and in their quality of relationships.^27^^,^^28^^,^^33^^,^^34^^,^^39^ Cho et al.^27^ observed that, for the wives, DC helped in accepting the possibility of separation, especially in the presence of behavioral problems.

Gústafsdóttir^37^ observed that the DC reinforced family ties, crucial to continue home care, and contributed to the contentment of life of the whole family.

The last benefit of the DC for the FC was the sharing of care with professionals, the information exchange and reflection on the case, and proposing care strategies.^32^^,^^35^^,^^38^^,^^39^ DC helped FC to develop a different perspective on the illness, as well as on its course, allowing a more respectful relationship regarding the individuality of the PwD.^37^

The three articles that studied the benefits of an integrated approach compared to standard care offered to PwD only, observed a significant decrease in FC suffering over PwD behavioral problems,^28^^,^^33^^,^^34^ increased feelings of competence, self-confidence and coping strategies to avoid problem situations and postponement of PwD institutionalization.^28^^,^^33^^,^^34^ The families enrolled in the program used more DC than families receiving standard care.^28^ Nevertheless, it was not effective for reducing the determinants of feeling of burden.^33^^,^^34^

The characteristics of the selected studies are shown in Table 1.

**Table 1:** Characteristics of studies selected.

Study	Objectives	Study design	Sample and setting	Assessment methods	Main results	MMAT- score
**Qualitative studies**						
Rokstad et al.^36^, 2017 Norway and Scotland	To explore and compare the experiences and outcomes of DC services designed for PwD as described by PwD and FC	Qualitative cross-national descriptive design	32 PwD-FC Dyads PwD N = 17 in Norway N = 19 in Scotland FC N = 17 in Norway N = 15 in Scotland	Qualitative interviews	**FC Outcomes** • Confidence in the staff, provided 'peace of mind' in the knowledge their relative was being well cared for • Benefit from a respite • In Scotland, FC felt they benefited from being able to stay and talk with staff. **PwD Outcomes** • Increased wellbeing • Increased social engagement • Improved functioning and mood • Improved relationships with FC • Overall satisfaction with the service.	****
Tretteteig^35^ et al., 2017 Norway	To provide an extended understanding of the situation of FC and examine to what extent DC can meet their need for support and respite	Cross-sectional Qualitative descriptive design	FC N =17	In-depth interviews	**FC Outcomes** • Relieves FC by meeting the PwD needs for social community, nutrition, physical activity, and structure and variety in everyday life • DC have a positive influence on the relationship between FC and the PwD • Led to a higher quality of time spent together and easier cooperation • Produced some hard feelings and challenging situations • DC gave the FC a feeling of freedom and increased the time available for their own needs, to be social and to work or do practical tasks undisturbed. • Provide the families with a sense of shared responsibility and relief while they are still active in their caregiving role. **PwD Outcomes** • DC reduces behavioural challenges • Contribute to the postponement of nursing home placement • This result depends on flexible opening hours, high quality of the DC program and regular cooperation with FC.	****
Liu et al.^23^, 2015 USA	To evaluate the magnitude of FC affective fluctuation and whether this is associated with caregivers' daily experiences, intervention, DC use that affects daily experiences by lowering exposure to care-related stressors, and with other caregiving characteristics	Cross-sectional Qualitative descriptive design	FC Users N = 173	Face-to-face interview; questionnaires; telephone interviews; Nonspecific Psychological Distress Scale; Daily Inventory of Stressful Events;	**FC Outcomes** • Changes the structure and routines of daily caregiving experiences • Provides temporary respite away from caregiving responsibilities • Relieves caregivers' exposure to potentially stressful behavior problems. • This recess prevents accumulation of daily negative affective reactions in both depression and anger. • It may help spouse or older caregivers or caregivers with lower education, and bring down their fluctuation in depression.	***
Zarit et al.^24^., 2014 USA	To examine the effects of use of DC by FC of PwD on daily stressors, affect, and health symptoms	Cross-sectional Qualitative descriptive design	FC Users N = 173	Daily telephone interviews; DRB; The Non-Specific Psychological Distress Scale; PANAS; ADL; IADL	**FC Outcomes** • Had lower exposure to care-related stressors on DC days, more positive experiences, more noncare stressors, lower anger and impact of noncare stressors on depressive symptoms.	****
Gústafsdóttir^37^ 2014 Iceland	To explore the collaboration between families and staff in day-care units caring for elderly subjects suffering from dementia.	Longitudinal Qualitative Interpretive phenomenological study	FC N = 8	Interviews	**FC Outcomes** • All families found the services of specialized day-care units both useful and pleasant • This resource made everyday life much more manageable for all persons involved.	***
Phillipson et al.^39^, 2012 Australia	To explore and understand DC use from the perspective of FC.	Longitudinal, qualitative	FC - N = 36 (29 users; 7 non-users); (25 spousal and 11 non-spousal).	Focus groups, semi-structured interviews, dyad interviews	**FC Outcomes** • They felt free to engage in other daily and social tasks, and to care for other family and friends. • They valued the opportunity to learn by observing team management strategies with PwD. The FC users had a more positive perception of DC than non-users. **PwD Outcomes** • The FC believes that PwD benefited from being engaged in positive occupation which enhanced their personhood or sense of self • Had gains from social interaction or mental stimulation. • The DC were perceived as improving the quality of life of the person with dementia.	***
de Jong et al.^32^, 2009 Netherlands	To study the needs and wishes of FC when providing skilled psychogeriatric DC.	Cross-sectional Qualitative descriptive design	N = 9 Dyads PwD / FC Co-residents N =4; Living alone N = 4 Nursing home N = 1	Semi-structured interviews and focus groups	**FC Outcomes** • The expertise of professionals and their relationship with the day-care users were considered important. • The FC need to know the structure and program of the DC • They expect the professionals to play a more active role in providing education, advice and support.	****
**Quantitative non-randomised studies**						
Logsdon^22^ et al., 2016 USA	To evaluate whether MCWS improved quality of life, mood, behavior, or functional status for PwD and whether FC experienced decreased stress, burden, or depression, compared with control dyads	Longitudinal Quasi-experimental, control group design	187 PwD-FC dyads Group of users N = 162; Controls non-users N = 25	MMSE; QOL-AD; ADL; IADL; RMBPC; CES-D; Screen for Caregiver Burden; PSS.	**FC Outcomes** • The MCWS caregivers exhibited significantly less distress over behavior problems, memory and depressive problems, than comparison caregivers. **PwD Outcomes** • No significant differences were seen between MCWS and comparison dyads at 3 months. After 6 months, MCWS participants exhibited significantly fewer depressive behaviors and other behaviors problems than comparison participants.	****
Kim et al.^25^, 2012 USA	To examine the effects of kin relationship on response to use of DC on feelings of burden, depressive symptoms, and positive affect for FC.	Longitudinal Quasi-experimental, control group design	FC N = 341 Users Group N = 182 (Wives = 67 + Daughters = 115) Controls non-users N = 159 (Wives = 64 + Daughters = 95)	Role overload; CES-D; PANAS; ADL; IADL; RMBP	**FC Outcomes** • Kin relationship affected the response to intervention • For depressive symptoms, both wives and daughters had lower scores over time compared to controls. • The wives and daughters had lower feelings of burden over time, but wives' scores dropped only to the level found among controls. • For positive affect, wives using DC showed a considerable decline over time.	***
Cho et al.^27^, 2009 USA	To examine whether kin relationship affects the timing of nursing home placement for caregivers who enroll a relative into an DC program compared with caregivers not using DC.	Longitudinal Quasi-experimental, control group design	FC N = 371 Group users N = 174 Wives 76 + Daughters 98 Controls non-users N = 197 Wives = 65 + Daughters = 132	In-person and telephone interviews; IADL; ADL; RMBPC; Role captivity and Role overload Scales; SCL-90; CES-D.	**FC Outcomes** • The DC can serve as a stepping stone for institutionalization. For wives, institutionalization was associated with feelings of overworking wives and behavioral problems of husbands • For daughters, the effect of using DC was to delay institutionalization	***
Mossello et al.^40^, 2008 Italy	To evaluate the effects of DC on cognitive, functional and behavioral status of PwD and on psychological well-being of FC, compared with nonrandomly matched controls receiving usual home care (HC)	Longitudinal Quasi-experimental, control group design	60 Dyads PwD/FC Group users N = 30 Controls non-users N = 30	NPI; CBI; MMSE; ADL; IADL; BDI	**FC Outcomes** • Burden significantly decreased compared to controls • No significant difference in between-group depressive symptoms change **PwD Outcomes** • The behavioural and psychological symptoms significantly decreased; • Reduction in psychotropic drugs prescription, whereas this increased in FC. • No significant difference in cognitive and functional changes	****
Droes et al.^33^, 2006 Netherlands	To retest the hypothesis that integrated comprehensive family support in the MSCP is more effective for reducing feelings of burden of FC and positively influencing some potential determinants of feelings of burden, than non-integrated support, such as regular DC. Explored the potential effect of the MCSP on the emotional impact that behavioural and psychiatric problems of the PwD have on FC.	Longitudinal Quasi-experimental, control group design	FC N = 84 Group MCSP users N = 71 Controls Day-Care users - N = 13	GHQ; SCS; JCS; Social Support list; Services list; LS; NPI; ASEP; CSDD; Behaviour Observation Scale for Intramural Psychogeriatrics; PGCMS	**FC Outcomes** • After seven months, no effect was found in psychological and psychosomatic symptoms or in the determinants of burden. • The integrated approach proved more effective than regular DC for decreasing psychological and psychosomatic symptoms in lonely FC. • The majority of carers experienced less burden and more professional support. **PwD Outcomes** • After seven months, significantly fewer persons were institutionalized as compared to the controls patients.	***
Gitlin et al.^28^, 2006 USA	To evaluate the short (3-month) and long-term (up to 12 months) effects of an innovative intervention, the Adult Day Services Plus (ADS Plus) program.	Longitudinal Quasi-experimental, control group design	N = 129 Dyads PwD/FC ADS Plus users N = 67 Controls DC users N = 62	Interviews; CES-D; ZBI; Memory and Problem Behaviors Scale; Perceived Change Index; REACH II	**FC Outcomes** • The ADS Plus Program provides evidence that DC is care which results in immediate and long-term clinically significant quality-of-life improvements for both the caregiver and impaired elder • Reduction in depressive symptoms • Increased confidence in managing troublesome behaviors • Enhanced overall perceived well-being	****
Droes et al.^34^, 2004 Netherlands	To compare whether the integrated support program (MCS) is more effective for reducing the feelings of burden of FC and positively influencing some determinants of burden experienced, such as behavioral changes, than the support offered by the regular DC.	Longitudinal Quasi-experimental, control group design	N = 55 Dyads PwD/FC Group MCS users N = 36 Controls DC users N = 19	CSQ; PGCMS; GHQ; LS; FCS; JCS; Social Support list; Services list; ASEP; BCRS; CSDD; Behaviour Observation Scale for Intramural Psychogeriatrics;	**FC outcomes** • There was no statistical indication that the MCS Program was more effective for reducing the feeling of overload of FC than the support offered by the regular DC • Participation in the program contributed to an increased sense of competence of FC and postponement of institutionalization.	***
Gaugler et al.^30^, 2003 USA	To determine whether DC use interacts with decreases in FC hours, to alleviate caregiver stress and negative mental health over time.	Longitudinal Quasi-experimental, control group design	FC N = 400 Group users N = 169 Controls non-users N = 231	In-person interviews; Role captivity; Role overload scale; CES-D; SCL-90; RMBPC, IADL, ADL	**FC Outcomes** The DC are potentially effective in restructuring caregiving time,providing FC with respite, when compared to non-users, thus leading to decreased feelings of exhaustion.	***
Zarit et al.^31^, 1998 USA	To evaluate the psychological benefits of DC for FC assisting a relative with dementia	Longitudinal Quasi-experimental, control group design	FC N = 324 Group users N =121 Controls non-users N = 203	CES-D; Role Captivity; Role Overload scales; Worry and strain; BSI; PANAS	**FC Outcomes** • The use of the day center significantly reduced overload, depression and anger compared to controls. **PwD Outcomes** • No decrease in behavioural problems.	***
Zank et al.^42^, 2002 Germany	To evaluate the effects of DC on PwD and FC	Longitudinal Quasi-experimental, control group design	PwD N = 83 Group users N = 43; Controls non-users N = 40 FC N = 62 Group users N = 40 Control non-users N = 22	LSQ; PSS; Self-esteem instrument; MADRS; MMSE; NAI; ADAS; NAO; Family Conflict Scale; Job-Caregiving Conflict Scale; ZBI; CES-D; RMBPC; semi-structured interview	**FC Outcomes** • No change in subjective well-being and burden between the groups in the longitudinal follow-up • On a semi-structured interview, the FC of PwD in the treatment group reported substantial positive change due to use of DC • High levels of satisfaction were reported **PwD Outcomes** • An improvement or stabilization of the subjective wellbeing and dementia symptoms in the treatment group in comparison with the control group • Follow-up data showed a significant decline in health in the control group in comparison with the day care users	****
**Quantitative descriptive**						
Kwok et al.^38^, 2013 Hong Kong	To examine the effectiveness of a dementia-specific DC	Longitudinal Quantitative descriptive design	90 Dyads PwD / FC N = 90	ZBI; MMSE; CMAI; IADL; BI; CDR; MFAC; BBS; MNA, PWI-ID; BMI	**FC Outcomes** • Dementia-specific DC reduced caregiver burden. **PwD Outcomes** • Maintained cognitive function and quality of life • Self-care ability, mobility and behavioural problems did not improve.	***
Zarit, et al.^26^, 2011 USA	To examine the daily assessment of FC on the impact of intervention and possible changes in the primary stressors associated with care, by comparing the days that the PwD were attended by the DC and the days they remained at home	Longitudinal Quantitative descriptive	FC - N = 121	Daily telephone interviews; DRB; in-home interview; MMSE; ADL; WRB	**FC Outcomes** • Total exposure to stressors and stress appraisals decreased significantly over time on • DC days compared with non-DC days. **PwD Outcomes** • Reduction in behavioral problems during the evenings was significant • Improved sleep immediately following DC use.	****
Mavall et al.^41^, 2007 Sweden	To evaluate whether the DC is an effective way of resting for caregivers residing and not-residing with their relative with dementia.	Longitudinal Quantitative descriptive design	FC N =51 Co-resident group [CR] N= 29; Non-co-resident group [NCR] N = 22	CES-D; Likert Scale.	**FC Outcomes** • Experienced a rest period • Were less worried and had decrease in care-related stress after four months • Non-co-residing FC whose relative continued in DC had significantly less burden, role captivity and depression than non-co-residing controls	****
Higgins, et al.^29^, 2005 USA	To evaluate whether a once-a-week activity-based DC program for dementia patients combined with 17 educational sessions for caregivers held at the same facility, over one year, increased quality of life (QOL), lowered levels of patient behavioral disturbance, and stimulated greater use of community-based resources	Longitudinal Quantitative descriptive design	Dyads PwD / FC Users - N = 21	MMSE; BRSD; QOL-AD; standardized questionnaire	**FC Outcomes** • Caregivers reported their own QOL as unchanged. **PwD Outcomes** • Cognitive function and behavioral symptoms worsened significantly Caregivers reported significant decrease in QOL for their loved ones, but patients reported essentially no change in QOL • The DC experience appeared to increase morale and decrease a sense of isolation for patients and caregivers alike.	***

Mini-Mental State Exam (MMSE); Revised Memory and Behavior Problem Checklist (RMBPC); Center for Epidemiological Studies Depression Scale (CES-D); Positive-Negative Affect Schedule (PANAS); Beck's Depression Inventory (BDI); Caregiver Burden Inventory (CBI); Assessment Scale for Elderly Patients (ASEP); General Health Questionnaire (GHQ); Carer Strain Questionnaire (CSQ); Loneliness Scale (LS); Feeling of Competence Scale (FCS); Jalowiec Coping Scale (JCS); Philadelphia Geriatric Centre Morale Scale (PGCMS); Montgomery and Asberg Depression Rating Scale (MADRS); Cornell Scale for Depression in Dementia (CSDD); Nuremberg Aging Observation Scale (NAO); Nuremberg Aging Inventory (NAI); Alzheimer's Disease Assessment Scale (ADAS); Life Satisfaction Questionnaire (LSQ); Brief Cognitive Rating Scale (BCRS); Neuropsychiatric Inventory (NPI); Symptom Checklist (SCL-90); Physical Self-Maintenance Scales (PSMS); Memory and Behaviour Problem Checklist (MBPC); Burden Interview (BI); Brief Symptom Inventory (BSI); Daily Record of Behavior (DRB); Weekly Record of Behavior (WRB); Quality of Life in AD scale (QOL-AD); Physical and Instrumental Self-Maintenance scales (ADL); Instrumental Activities of Daily Living (IADL); Perceived Stress Scale (PSS); Zarit Burden Inventory (ZBI); Cohen-Mansfield Agitation Inventory (CMAI); Clinical Dementia Rating (CDR); Modified Functional Ambulation Category (MFAC); Berg Balance Scale (BBS); modified Barthel Index (BI); Mini Nutritional Assessment (MNA); Personal Wellbeing Index-Intellectual Disability (PWI-ID); Body Mass Index (BMI); National Institutes of Health multisite Resources for Enhancing Alzheimer's Caregivers' Health (REACH II); CERAD Behavior Rating Scale for Dementia (BRSD).

### Configuration of day care

***Sources of subsidy.*** Different sources of subsidy for DC were reported. Rokstad et al.^36^ stated that in Norway, when a DC is considered a health care service for the PwD, it is regulated and subsidized by the municipalities. If it is defined as a respite for the FC there is a subsidized payment for attendance. In Scotland, the DC is provided by statutory, private, and third sector organizations and regulated by the Care Inspectorate. The Dutch skilled DC facilities are part of the nursing home organization, and are subsidized and legislated by the government.^32^^-^34 In Germany, these services are paid for by health insurance, care insurance, patients, or social welfare.^42^ In New Jersey (USA), the statewide network of adult DC is provided in part by the Department of Health and Senior Services for FC with low and moderate income.^26^^,^^31^ One study was financed by the U.S. Administration on Aging Alzheimer's Disease Demonstration Grants to States funds and state matching funds.^22^ Three studies were conducted in DC funded by private sector and third sector or self-funded partnerships without subsidies or legislation by the government.^29^^,^^38^^,^^41^ Eleven articles did not state whether the DC was funded by an institution or whether it was paid for by the users.^23^^,^^24^^,^^27^^,^^28^^,^^30^^,^^35^^,^^37^^,^^39^^,^^40^

***Structure.*** Twelve articles provided no information about the structure of the DC.^23^^-^25^,^^27^^,^^28^^,^^30^^,^^35^^-^39^,^^41^ Nine studies presented information on the regimen of use of the DC, which varied from one to five times a week and from 4 to 8 hours daily.^22^^,^^26^^,^^29^^,^^31^^-^34^,^^40^^,^^42^ Regarding the competence of the team, only six studies reported having a multi-professional staff,^22^^,^^28^^,^^32^^,^^36^^,^^40^^,^^42^ which included social assistants,^22^ nurses,^22^^,^^28^^,^^40^^,^^42^ occupational therapist and speech therapist,^22^ physiotherapist and geriatrician,^40^ Director of center, family service provider, activities specialist,^28^ and three reported having the participation of volunteers.^29^^,^^36^^,^^42^

***Program.*** Eleven studies did not describe the activities offered.^23^^-^25^,^^27^^,^^28^^,^^30^^,^^31^^,^^35^^,^^37^^,^^39^^,^^41^ Two studies reported recreational activities, social interaction and meals.^36^^,^^40^ In addition to these activities, seven studies also included psychosocial activities, activities of daily living, cognitive stimulation, physical and psychomotor activity, and music therapy.^22^^,^^26^^,^^29^^,^^32^^,^^33^^,^^34^^,^^38^^,^^42^

Twelve articles made no mention of whether the DC offered caregiver support programs.^23^^-^27^,^^30^^-^32^,^^35^^,^^36^^,^^39^^,^^41^ Caregivers were supported by designated case managers in one study,^38^ informal counseling was provided in another,^40^ two studies offered educational program for caregivers,^29^^,^^42^ and one offered caregiver support groups.^37^ Logsdon et al.^22^ described the Memory Care and Wellness Services (MCWS) program. This program provides specialized activities and exercise for PwD. The FC are involved in planning care for PWD and receive support and information, including referral services.

Drões et al.^33^^,^^34^ evaluated whether an integrated program offered to the dyad would positively influence some potential determinants of burden. In the program, the PwD participated in social activities, recreation and rehabilitation. Simultaneously, the FC received emotional and social support based on a psychosocial diagnosis. The program included ten educational groups, a biweekly support group. PwD and FC participated in a weekly counseling meeting and a monthly meeting with all staff in which they discussed possible adjustments to the treatment program.

Gitlin et al.^28^ studied Adult Day Services Plus (ADS Plus), a special support program for FC focused on user needs. FC were interviewed at the beginning of treatment, after three months and at one year. The FC received advice, information and educational materials through periodic personal contact or by telephone. At the same time, the PwD regularly participated in the DC program of activities.

***Objectives and therapeutic proposals.*** The DC proposes to offer significant activities,^36^^,^^41^ a safe environment, as well as social, emotional and health support^22^^,^^29^^,^^32^^,^^36^ for the PwD. Some programs offer rehabilitation care,^32^ recreational activities,^36^^,^^40^ and motor and cognitive stimulation.^26^^,^^28^^,^^32^^-^34^,^^36^^,^^38^^,^^42^ They propose to improve their coping strategies^36^ and quality of life,^36^^,^^38^ maintain cognitive functioning, physical ability, functional capacity.^26^^,^^28^^,^^32^^-^34^,^^36^^,^^38^^,^^42^ behavioral status, nutritional status.^38^ as well as promote a sense of kinship^29^ and well-being.^42^ The DC programs also serve as a respite service for FC.^22^^,^^33^^,^^34^^,^^36^^,^^40^ The program objectives are to alleviate FC stress and burden,^40^^,^^42^ improve their knowledge about the disease^28^^,^^33^ and sense of competence related to the caregiving tasks,^33^ to give them emotional and social support,^28^^,^^33^^,^^41^ offer counseling,^28^ facilitate continued care at home^22^^,^^40^ and postpone the need for institutionalization.^36^^,^^41^

***Theoretical basis.*** Only four studies reported the theoretical basis of the support program offered to FC. Drões et al.^33^^,^^34^ cited the Meeting Centres Support Program (MCSP), with a theoretical framework based on the adaptation coping model^43^^,^^44^ and the model of determinants of experienced burden. The models consider negative social circumstances as determinants of burden. Gitlin et al.^28^ stated that the ADS Plus was based on the Pearlin et al.^46^ stress process model. The program targets the primary stressors of caregiving: behavior problems of the PwD and the physical, mental, and social health of the FC. The approach was used to support positive intrapsychic factors (such as the perception of competence and rewards of caregiving), as well as to introduce positive coping strategies (such as problem solving) in order to mediate objective stressors of caregiving. Zank et al.^42^ only reported that the DC units studied offered a rehabilitation program based on clinical experience and gerontological knowledge.

The structure, objectives and theoretical basis of the DC are depicted in Table 2.

**Table 2:** Characteristics of day care in studies selected.

Study	DC setting	Objectives of day care services	Design of interventions	Theoretical principles	Effectiveness indicators used
**Rokstad et al.^36^, 2017**	Subsidy: Norwegian - DC is provided and regulated by the municipalities and run mainly by local authorities that must offer in-home nursing care and residential care. The service is free of charge if considered a health care service for the PwD. If defined as a respite service for FC, there is a subsidized payment for attendance. Scotland: DC is provided by statutory, private and third sectors organizations. There may also be some direct costs to attendees for lunch or transport. All social care services are regulated by the Care Inspectorate. Team: Norwegian - The staff comprise paid health and social care workers, but some DC include volunteers in the paid staff. Scotland - there is a mix of paid professional staff and volunteers Regularity: Not described	# To offer meaningful activities and social support and a safe environment to enhance coping and improve quality of life for users # DC should act as a respite service and possibly postpone the need for nursing home placement.	PwD: The day is structured with repeated routine centered on conversations, mealtimes, activities (singing, physical games and exercising, arts and crafts, word games, and quizzes) with an emphasis on fun and humor.	Not described	# Confidence in the staff # Increased social interaction, wellbeing, social engagement, improved function and mood, and relationships with caregivers # Quality is dependent on the way staff organized the service and engaged with people.
Tretteteig et al.^35^, 2017	Subsidy: Not described Regularity: for approximately 2 - 18 months, 2 - 5 days a week.	Not described	Not described	Not described	
Logsdon^22^ et al., 2016	Subsidy: Not described Team: including social services, nursing, and occupational/speech therapy. Staffing ratio = 1:4 Regularity: A program operating at least 2 days a week, and at least 5 hours per day	# To facilitate continued care at home # To provide respite for the caregiver, as well as emotional support # To deliver health support for the PwD	FC: Involvement of the caregiver in care planning; Caregiver support, including information and referral services PwD: A program of specialized activities and exercise	Not described	# Better health, mood # Fewer behavioral problems # Better quality of life (QOL);
Liu et al.^23^, 2015.	Not described	Not described	Not described	Not described	Not described
Zarit et al.^24^, 2014	Not described	Not described	Not described	Not described	
Gústafsdóttir^37^, 2014	Not described	Not described	FC: Caregiver support groups. They talk regularly to the head of the DC or to the unit nurse and interact with everyone in the DC when they attend events such as travel or dance sessions.	Not described	
Kwok et al.^38^, 2013	Subsidy: A self-financed dementia-specific DC Regularity: Participants attended its respite service and also stayed in their own home at least 1 day per week.	# To alleviate caregivers' stress and burden # To maintain cognitive functioning, physical ability, daily functional ability, behavioural status, nutrition level, and quality of life of PwD.	FC: Supported by designated case managers PwD: Group training on memory, activities of daily living, reality orientation, and cognitive stimulation.	Not described	Not described
Kim et al.^25^, 2012	Subsidy: Funded in part by a statewide subsidy available to family members caring for a PwD Regularity: two days per week or more	Not described	Not described	Not described	Not described
Phillipson et al^39^, 2012	Not described	Not described	Not described	Not described	Not described
Zarit et al.^26^, 2011	Regularity: three days a week, 6 hours on the program, not counting travel time	Not described	PwD: Daily routines, physical activities, social activities, cognitive stimulation	Not described	# Decreased frequency of behavioral problems # Reduced FC exposure to stress and its negative consequences
de Jong et al.^32^, 2009	Subsidy: the government covers the costs Team: multidisciplinary professional staff Regularity: two to four days each week, 7 hours a day Transportation: included	# To provide information at client level/cooperation Mutual familiarity # To promote trust and flexibility in schedule # To give information and advice # To offer specialized medical and paramedical care # To offer physical and therapeutic activities aimed at rehabilitating # To run individualized activities	PwD: Routinely provide systematic assessment, rehabilitation, education and support activities	Not described	# Identification of early warning of problems or changes in health, offering a guarantee of crisis intervention # Increased dignity and self-esteem # Offering of flexibility in relation to schedules by adjusting the service according to the conditions of each PwD # Interaction, communication, information and support offered by the team # Good team relationship with caregivers and users # Staff should inform FC about the experiences of PwD
Cho et al.^27^, 2009	Subsidy: network of DC programs and subsidies provided to help families pay for it	Not described	Not described	Not described	Not described
Mossello et al.^40^, 2008;	Team: multi-professional team, including nurses and physiotherapist, supervised by a geriatrician Regularity: 2 to 6 days weekly for 8 hours a day	# To stimulate residual cognitive abilities # To reduce BPSD	FC: Informal counseling for caregivers PwD: individual care plans, based on geriatric multidimensional assessment of occupational and recreational activities Controls: Usual home care.	Not described	# Environmental management # Providing relief for caregivers and reducing their stress # Keeping patients close to their familiar environment # Decrease in behavioral changes
Mavall et al.^41^, 2007	Not described	# To support FC # To provide a meaningful day # To delay institutionalization	Not described	Not described	# Decrease in time that FC spends caring and increase in time FC can dedicate to their other needs. # Decrease in depression and somatic problems of FC. # Improvement in psychological well-being of caregiver;
Droes et al.^33^, 2006	Regularity: FC: Ten informative meetings bi-weekly discussion group PwD: Three days a week Dyad: A monthly centre meeting A weekly consultation hour	# To support FC, improve their knowledge and sense of competence and support them emotionally and socially. # To offer some respite	FC: Participate in the discussion group whenever they feel the need to do so. PwD: Offer recreational and social activities for reality orientation training, reminiscence, validation, psychomotor therapy and music therapy. DYAD: Can utilize weekly consulting and participate in social festivities and excursions. Support and case management is provided by professional staff. A collaboration protocol regulates cooperation between the staff and the professional care and welfare services in the neighborhood involved with PwD. Controls: PwD participated in the regular DC. If necessary, medical care, physiotherapy or occupational therapy are also available. The FC is only marginally involved.	The adaptation coping model^40^^,^^41^; The model of determinants of experienced burden of carers of PwD	# Reduction of psychological and psychosomatic symptoms in carers who feel more lonely # Increase in sense of competence for FC of PwD who exhibit behavioral problems # Increase in satisfaction with FC coping strategies # Reduction of loneliness in carers older than 75. # Decrease in emotional impact that behavioural and psychiatric problems of the PwD have on the carer.
Gitlin et al.^28^, 2006	Subsidy: Uses existing DC staff and resources Team: center director, family service provider, activity specialist, nurse, and program assistants, with a direct staff/elder ratio of 1:5. Regularity: FC received an average of 1 hour per month of contact with the service.	# ADS Plus integrates care management into regular DC to address the specific concerns and needs of FC and has the purpose of providing emotional support, counseling, education, and referral targeting the specific concerns identified.	FC: Meet face-to-face with the site service Director in order to: (a) identify areas of concern and needs; (b) develop a care plan for areas of difficulty; (c) implement an agreed-upon care plan with four components: counseling, education, referral, and periodic supportive contact with the service Director. Subsequently, the service Director meets with FC either face-to-face when their relative visits the center, or by telephone. Additionally, the service Director provides targeted education materials through mailings. The purpose of each follow-up contact is to provide emotional support, counseling, education, and specific referrals for concerns identified. PwD: Receive usual DC support and the ADS Plus Program for the caregivers. Controls: PwD use of DC; the FC have access to staff to obtain information as needed. The contact is generally not programmed or purposeful, nor is the structured care management service. Support and educational groups are occasionally offered.	The stress process model^42^	# Quality-of-life improvements for both the FC and PwD # Reduction in depressive symptoms # Increased confidence in managing troublesome behaviors # Enhanced overall perceived well-being.
Higgins, et al.^29^, 2005	Subsidy: The University of Texas Southwestern Medical Center and the Greater Dallas Chapter of the Alzheimer's Association Team: volunteer staff Regularity: once a week for 6 hours a day	# To provide respite and education for FC. # To provide an inviting, comfortable, and stimulating environment for PwD	FC: Educational program for caregivers PwD: Use of overlearned skills in simple games and stimulation of recent memory, associative processes, and simple socially appropriate motor tasks	Not described	# Increased QOL in both PwD and FC # Lower levels of behavioral symptoms # Improvement in use of community resources # Improvement in educational experience for FC.
Dröes et al.^34^, 2004	Regularity: FC: Ten informative meetings and bi-weekly discussion group PwD: Three days a week and a weekly consultation hour Dyad: A monthly centre meeting	# To support FC, improve their knowledge and sense of competence and to support them emotionally and socially. # To offer some respite	FC: Informative meetings and long-term discussion group. PwD: Uses the DC activity club, which organizes creative recreational activities and participates in psychomotor therapy. A consultation hour organized, where PwD and FC can get personal advice. A meeting during which the dyad and professionals express their wishes concerning changes in the program. Controls: FC have incidental contacts with social worker and other staff members. PwD participated in the DC program.	The adaptation coping model^40^^,^^41^	# Reduction in the feelings of burden of FC; # Delaying admission in a nursing home # Positively influencing some potential determinants of burden.
Gaugler et al.^30^, 2003	Not described	Not described	Not described	Not described	Not described
Zank et al.^42^, 2002	Subsidy: health insurance, care insurance, patients, or social welfare. Team: geriatric nurses, and untrained young men doing community work instead of military service. Transport: Provided Regularity: at least twice a week plus usual home-based community care 7 days per week, up to five times daily	# To enhance patients' well-being and competence # To reduce loneliness and depressive symptoms # To reduce burden placed on caregivers.	FC: Sometimes informative groups for caregivers are offered. PwD: DC plus usual home-based community care. The DC offer structured daily routine, individual training of residual competencies, new social contacts and constructive activities. The program consists of everyday life activities, games, individual training, and outings, tailored to the individual patient with his or her unique biography, specific weaknesses and strengths. Controls: PwD receiving only home-based care	Rehabilitation program based on clinical experience and gerontological knowledge	Improvement in emotional well-being and ADL of PwD. Reduction in primary stressors, the stress and burden of FC and increase in their free time; Provision of information about the disease; Reduction in family conflict and conflict between job demands and perceived caregiving demands.
Zarit et al.^31^, 1998	Regularity: 5 days a week for 7 hours per day. Many centers extend hours and a few have weekend hours to accommodate FC Transport: Provided	Not described	Not described	Not described	Not described

## DISCUSSION

Historically, the DC, as a strategy for supporting FC, was originally intended to offer a break in caregiving tasks, and to maintain or improve the health and quality of life of the PwD-FC dyad. This concept of service was inspired by studies on the burden and coping strategies of family caregivers and the impact on their quality of life.^2^^-^7^,^^9^ Currently, DC are also considered a fundamental community-based technology for health care, which aim to offer all interventions to promote health, prevent and treat, improve rehabilitation and long-term care.^4^^-^6^,^^35^

The need for support, together with the benefits outlined, has justified investment from different actors, such as governments, statutory, third sector organizations, health insurance and assistance, universities, private institutions and families.^25^^,^^29^^,^^31^^-^34^,^^36^^,^^38^^,^^41^^,^^42^ Logsdon et al.^22^ reported that DC is the least expensive alternative for providing rest, emotional and health support to PwD, when compared to a non-medical home health intervention, home care or home nursing care. Although there is some evidence of the effectiveness of DC, the heterogeneity in service settings, activity schedules and staff, do not allow solid cost-effectiveness conclusions.^2^^,^^6^^,^^22^^,^^44^ This factor also contributes to limitations in funding and availability of these programs.^2^^,^^6^^,^^22^^,^^44^

Quantitative approaches with controls to evaluate the impact of this care^22^^,^^25^^,^^27^^,^^30^^,^^31^^,^^40^^,^^42^ and qualitative studies, to ascertain the expectations, needs and beliefs^32^^,^^35^^,^^39^ and level of satisfaction^36^ of the FC were implemented. No randomized studies were found, and some studies questioned the validity of this type of design due to ethical concerns with the impact of non-access to treatment for the controls in the case of longitudinal studies.^26^^,^^45^

There was methodological heterogeneity in the characteristics of the FC samples and in the assessments used. This diversity hinders comparison among studies and their outcomes. For example, case studies with controls compared the days of DC treatment to the days when the PwD was cared for at home,^23^^,^^24^^,^^27^^,^^30^^,^^31^ FC of PwD users were compared to non-users,^25^^,^^39^ while others only interviewed FC of DC users.^35^^,^^37^^,^^39^ Despite the great diversity of methods, sample sizes, measuring instruments and interviews, most of the studies reported similar positive results with DC use.^22^^-^37^,^^39^^-^42 The same heterogeneity was also evident in the configuration of the intervention regarding three aspects: the structure and organization of the DC programs, the design, and the theoretical basis.

### Structure and organization

Attendance frequency varied from one to five times a week and the number of hours from four to eight hours per day.^22^^,^^26^^,^^29^^-^34^,^^40^^,^^41^ This variability possibly influences the results presented in the studies. Research on the needs and expectations of FC has identified an important demand for flexibility of the DC in relation to available days and times, and the possibility of a regular or intermittent frequency,^7^^,^^9^^,^^28^^,^^32^^,^^35^^,^^36^^,^^39^ numbering among the factors determining users' level of satisfaction. These aspects involve accommodating possible changes in the daily needs and schedules of FC. This flexibility has a considerable influence on the levels of burden and quality of life of the dyad.^3^^,^^9^^,^^28^^,^^32^^,^^35^^,^^36^^,^^39^

### Intervention design

The diversity of the intervention designs impacts the results of the studies.^35^ Some studies did not describe the activities offered, mainly because they aimed to investigate the benefit of the break from care for the FC.^23^^-^25^,^^27^^,^^30^^,^^31^^,^^35^^,^^37^^,^^39^^,^^41^Among the programs described, some offered activities for PwD only,^26^^,^^32^^,^^36^ while others included activities for the dyad.^22^^,^^28^^,^^29^^,^^33^^,^^34^^,^^37^^,^^38^^,^^40^^-^42

The two approaches, including the caregivers in the service or otherwise, identified different protocols of activities aimed at PwD. Programs of recreational activities with an emphasis on socialization and well-being,^36^^,^^40^ and others that included both motor and cognitive stimulation plus rehabilitation.^26^^,^^28^^,^^32^^-^34^,^^36^^,^^38^^,^^42^ The results showed that the provision of physical, cognitive and social stimuli positively impacted well-being and health in general, sleep quality, functioning and cognitive readiness, even in participants with reduced cognitive capacity.^26^^,^^35^^,^^36^^,^^38^^,^^40^^,^^42^ These studies reinforce the premise that lack of activity contributes to the emergence of PwD neuropsychiatric symptoms.^46^^-^50 Conversely, a multimodal program of cognitive and physical rehabilitation can improve socialization mood and also reduce caregiver burden.^46^^-^50

The results concerning the impact of activities on behavioral changes were contradictory. Zarit et al.^26^ and Mossello et al.^40^ observed a significant reduction in behavioral changes, leading to an improvement in sleep in users on days of DC use,^26^ along with a reduction in the prescription of psychotropic drugs, when compared with non-users of DC.^40^ Kwok et al.,^38^ on the other hand, observed no improvement. These disparities may be related to methodological differences and to intervention time. In any case, the improvements observed reduced the impact of primary stressors on FC and, as a secondary result, burden was reduced.^36^^,^^38^^,^^39^ In turn, the certainty that the PwD would be protected in a friendly environment, involved in meaningful and stimulating activities, and would receive personalized attention adapted to their interests and levels of functionality, were relevant aspects for FC confidence in the service.^32^^,^^35^^,^^36^^,^^39^ Notably, the high degree of heterogeneity precluded analysis of the effect size of the studies.

Different approaches to FC were observed. In one study, the DC program offered informal counselling,^40^ but the model was not described. In some programs, the FC were only marginally involved in care.^28^^,^^34^^,^^42^ In others, the program included support interventions,^22^^,^^28^^,^^29^^,^^33^^,^^34^^,^^37^^,^^38^ such as educational programs^22^^,^^28^^,^^29^^,^^33^^,^^34^^,^^42^ and support groups,^22^^,^^28^^,^^33^^,^^34^^,^^37^ both with the aim of improving FC sense of competence regarding the caregiving tasks. Some programs also offered social activities, such as parties and trips, during which the FC had the opportunity to interact in a fun way with their family member.^33^^,^^34^^,^^37^ In some services, the FC were supported by case managers or had regular meetings with staff.^22^^,^^28^^,^^37^^,^^38^ In such cases, FC, along with staff, analyzed needs, and developed a care plan to be implemented both at home and at the DC.^22^^,^^28^^,^^33^^,^^34^^,^^38^ Treatment follow-up occurred through face-to-face meetings or by telephone contact, in which FC expressed their observations, concerns and needs, received counseling and were informed about referral services.^22^^,^^28^^,^^33^^,^^34^^,^^37^^,^^38^ The way in which DC met the need for flexibility, support, information and sharing of responsibilities was positively highlighted by FC.^22^^,^^28^^,^^32^^-^36^,^^42^ The studies suggested that this integrative approach better addresses the different needs of FC.^28^^,^^33^^,^^34^ However, these results cannot be considered conclusive since they are similar to those observed in other non-integrative approaches. In addition, the heterogeneity observed in DC approaches hinders characterization of a gold standard for this kind of intervention.

### Theoretical basis

With regard to theoretical background, the adaptation coping model adopted by the MCS was briefly described in the two articles by Drões et al.^33^^,^^34^ This model provides a framework for explaining behavioral problems in PwD, based on the principle that people constantly strive for balance when confronted with changes in their existence.^43^^,^^44^ The socioenvironmental, material and determinant factors of the disease are believed to interfere in the subjective evaluation of the PwD about their condition. Consequently, given the imbalance generated by the disease, the person has to carry out cognitive, social and emotional adaptive tasks. This adaptation implies a learning process and depends on the quality of interaction with the environment.^43^^,^^44^ Therefore, the dyad are assisted in this complex process of adaptation that includes learning to deal with limitations and dependence, maintaining and establishing social relations, dealing with environmental aspects and prescribed treatments, preserving an emotional balance, a positive self-image and preparing for an uncertain future.^43^^,^^44^ This theory offers an understanding of the PwD response to the illness and guides a stimulation program that facilitates the adaptive process and support that helps the FC understand the condition of the family member and to reflect on more effective care strategies. However, by focusing only on the aspects related to primary stressors, it disregards possible secondary stressors that interfere with the care relationship and contribute to the feeling of burden. The focus is on understanding the PwD experience, the consequences of the illness, and possible coping strategies.

The stress process model of Pearlin et al.^45^ was cited by Gitlin et al.^28^ as the theoretical basis of ADS Plus. Firstly, this theory considers the difficulties and problems anchored directly in care as primary stressors. Secondly, the socioeconomic conditions, conflicts and tensions resulting from working, family relationships and social life, or intrapsychic tensions associated with the personality or decrease in the caregiver's self-concept were considered secondary stressors.^45^ From this perspective, coping and social support can potentially influence multiple aspects throughout the stress process.^45^ This theory offers a theoretical scope that allows a diagnosis and understanding of the situation of the FC, and proposes specific intervention strategies to support and improve the resources and conditions of care. The focus of the intervention was centered on FC experience and their resources for care.

The person-centered approach was cited as the recommended theoretical axis to guide the care model of the DC.^36^ This theory entails a theoretical principle underlying the different psychosocial models as it shifts the focus of the disease to the PwD and the way in which they experience their condition.

These approaches are offered as theoretical references for care delivery and are part of the set of psychosocial models used in the field of Psychogeriatrics.^44^ In this field, the theories articulate three fundamental axes: the focus on the sick person and their experience, the focus on the caregiver and their experience, besides understanding of the disease and its consequences.^44^ The approaches seek to understand the multidimensional aspects that contribute to the condition of the PwD and FC, considering their needs, suffering, resources, coping strategies and the impact of illness on the family relationship. The theories analyzed are not exclusive, as each focuses on fundamental aspects for the provision of dementia care. The development of further DC should include a theoretical framework in order to enable more consistent intervention.

### Limitations

The possible influence of socioeconomic and cultural differences between the samples studied calls for caution in the interpretation of results. Likewise, the lack of standardization in the configuration of DC hampers comparison of results. In addition. the conception of DC as a rest service, restricts the understanding of its therapeutic role as a treatment and rehabilitation device for PwD, and a source of guidance and support for FC.

Most of the studies failed to describe the strategies of interaction, support and guidance for FC, and did not explain the theoretical framework that conceptually defines the services model and its therapeutic goals. The lack of a detailed description limits the interpretation of the congruence of results in relation to objectives proposed and intervention programs.

In conclusion, The reduction in daily exposure of the FC to primary stressors promoted a rest period and contributed to the continuity of the family living together with the PwD, delaying institutionalization. The DC appears to be a promising technology for reducing FC burden, thereby improving their health and quality of life.

Integrated programs that cater for the needs of the PwD and their FC represent a promising approach that meets their needs and demands in a multidimensional manner. The DC is a technology that aims to promote health, prevent disease and also provide treatment through interventions that minimize burden and enable long-term care. However, there is a lack of integrative approaches that articulate the contribution of different complementary theories and guide comprehensive and cost-effective intervention programs.

## References

[1] Prince M, Wimo A, Guerchet M, Ali GC, Wu Yutzu, Prina M. World Alzheimer Report 2015. The global impact of dementia: an analysis of prevalence, incidence, cost and trends. London: Alzheimer's Disease International; 2015.

[2] 2 Alzheimer's Disease International and WHO. Dementia: a public health priority. Geneva: World Health Organization; 2012 (http://www.who.int/mental_health/publications/dementia_report_2012/en/, accessed 8 March 2017).

[3] 3 The Technical Report of European Collaboration on Dementia (Eurocode) (2006-2008). www.alzheimer-europe.org and www.dementia-in-europe.eu

[4] Bartfay E, Bartfay WJ. Quality-of-Life Outcomes Among Alzheimer's Disease Family Caregivers Following Community-Based Intervention. West J Nurs Res. 2013;35(1):98-116. 10.1177/0193945911400763 21415243

[5] Vandepitte S, Noortgate NVD, Putman K, Verhaeghe S, Verdonck C, Annemans L. Effectiveness of respite care in supporting informal caregivers of persons with dementia: a systematic review. Int J Geriatr Psychiatry 2016;31:1277-88. 10.1002/gps.4504 27245986

[6] Mason A, Weatherly H, Spilsbury K, Arksey H, Golder S, Adamson J, et al. A systematic review of the effectiveness and cost-effectiveness of different models of community-based respite care for frail older people and their carers. Health Technol Assess. 2007;11(15):1-157, iii. Review. 10.3310/hta11150 17459263

[7] Tretteteig S, Vatne S, Rokstad AMM. The influence of day care centres for people with dementia on family caregivers: an integrative review of the literature. Aging Ment Health. 2016;20(5):450-62. 10.1080/13607863.2015.1023765 25815563

[8] Fields NL, Anderson KA, Dabelko-Schoeny H. The effectiveness and adult day services for older adults: A review of the literature from 2000 to 2011. J Appl Gerontol. 2014;33(2):130-63. 10.1177/0733464812443308 24652952

[9] Colvez A, Joel ME, Ponton-Sanchez A, Royer AC. 2002. Health status and work burden of Alzheimer patients' informal caregivers: comparisons of five different care programs in the European Union. Health Policy 2002;60(3):219-33. 10.1016/s0168-8510(01)00215-9 11965332

[10] Santos RL, Souza MFB, Brasil D, Dourado M. Group interventions focused on the burden of caregivers of patients with dementia: A systematic review. Rev Psiq Clin. 2011;38(4):161-7.

[11] Pot A. Caregivers' perspectives. A longitudinal study on the psychological distress of informal caregivers of demented elderly. Academic thesis: Vrije Universiteit, Amsterdam; 1996.

[12] Kramer BJ, Vitalino PP. Coping: a review of the theoretical frameworks and the measures used among caregivers of individuals with dementia. J Gerontol Social Work. 1994;23:15-174.

[13] Lezak MD. Living with the characterologically altered brain-injured patient. J Clin Psychiatry. 1978;39:592-8. 681289

[14] Zarit SH, Stephens MA, Townsend A, Greene R, Femia EE. Give day care a chance to be effective: A commentary. J Gerontol B Psychol Sci Soc Sci. 2003;58(3):P195-6; discussion P197-9. 10.1093/geronb/58.3.p195 12730312

[15] Femia EE, Zarit SH, Stephens MA, Greene R. Impact of adult day services on behavioral and psychological symptoms of dementia. Gerontologist. 2007;47(6):775-88. 10.1093/geront/47.6.775 18192631

[16] Zarit SH, Kim K, Femia EE, Almeida DM, Klein LC. The effects of adult day services on family caregivers' daily stress, affect, and health: Outcomes from the daily stress and health (DaSH) study. Gerontologist. 2014;54(4):570-9. 10.1093/geront/gnt045 PMC4155447 23690056

[17] 17 Tarrant S. 2010 Next Steps Think Tank: Adult day services. Retrieved from http://www.ageandcommunity.org/products.attachment/2010adultdaythinktank2667/2010AdultDayThinkTank.pdf

[18] Moher D, Liberati A, Tetzlaff J, Altman DG. PRISMA Group. Preferred reporting items for systematic reviews and meta-analyses: the PRISMA statement. PLoS Med. 2009;6(7):e1000097. 10.1371/journal.pmed.1000097 PMC2707599 19621072

[19] Brasil. Ministério da Saúde. Secretaria de Ciência, Tecnologia e Insumos Estratégicos. Departamento de Ciência e Tecnologia. Diretrizes Metodológicas: elaboração de revisões sistemáticas e metanálise de estudos observacionais e comparativos sobre fatores de risco e prognóstico/Ministério da Saúde, Secretaria de Ciência, Tecnologia e Insumos Estratégicos, Departamento de Ciência e Tecnologia. - Brasília: Ministério da Saúde; 2014:132.

[20] 20 Pluye P, Robert E, Cargo M, Bartlett G, O'Cathain A, Griffiths F, et al. (2011). Proposal: A mixed methods appraisal tool for systematic mixed studies reviews. Department of Family Medicine, McGill University, Montreal, Canada. Retrieved on [date] from http://mixedmethodsappraisaltoolpublic.pbworks.com. Archived by WebCite® at http://www.webcitation.org/5tTRTc9yJ

[21] Pace R, Pluye P, Bartlett G, Macaulay AC, Salsberg J, Jagosh J, Seller R.Testing the reliability and efficiency of the pilot Mixed Methods Appraisal. Tool (MMAT) for systematic mixed studies review. Int J Nurs Stud. 2012;49(1):47-53. 10.1016/j.ijnurstu.2011.07.002 21835406

[22] Logsdon RG, Pike KC, Korte L, Goehring C. Memory Care and Wellness Services: Efficacy of Specialized Dementia Care in Adult Day Services. Gerontologist. 2016;56(2):318-25. 10.1093/geront/gnu012 24615230

[23] Liu Y, Kim K, Almeida DM, Zarit SH. Daily Fluctuation in Negative Affect for Family Caregivers of Individuals With Dementia. Health Psychology. 2015;34(7):729-40. 10.1037/hea0000175 PMC5533950 25365414

[24] Zarit SH, Kim K, Femia EE, Almeida DM, Klein LC. The Effects of Adult Day Services on Family Caregivers' Daily Stress, Affect, and Health: Outcomes From the Daily Stress and Health (DaSH) Study. Gerontologist. 2014;54(4):570-9. 10.1093/geront/gnt045 PMC4155447 23690056

[25] Kim K, Zarit SH, Femia EE, Savla J. Kin relationship of caregivers and people with dementia: stress and response to intervention. Int J Geriatr Psychiatry. 2012;27:59-66. 10.1002/gps.2689 21322030

[26] Zarit SH, Kim K, Femia EE, Almeida DM, Savla J, Molenaar PC. Effects of adult day care on daily stress of caregivers: a within-person approach. J Gerontol B Psychol Sci Soc Sci. 2011;66:538-46. 10.1093/geronb/gbr030 PMC3155027 21642593

[27] Cho S, Zarit SH, Chiriboga DA. Wives and daughters: the differential role of day care use in the nursing home placement of cognitively impaired family members. Gerontologist. 2009;49:57-67. 10.1093/geront/gnp010 PMC2664617 19363004

[28] Gitlin LN, Reever K, Dennis MP, Mathieu E, Hauck WW. Enhancing quality of life of families who use adult day services: Short- and longterm effects of the adult day services plus program. Gerontologist. 2006; 46(5):630-9. 10.1093/geront/46.5.630 17050754

[29] Higgins M, Koch K, Hynan LS, Carr S, Byrnes K, Weiner MF. Impact of an activities-based adult dementia care program. Neuropsychiatr Dis Treat. 2005;1(2):165-9. 10.2147/nedt.1.2.165.61050 PMC2413197 18568062

[30] Gaugler JE, Jarrott SE, Zarit SH, Stephens MA, Townsend A, Greene R. Adult day service use and reductions in caregiving hours: Effects on stress and psychological wellbeing for dementia caregivers. Int J Geriatr Psychiatry. 2003;18(1):55-62. 10.1002/gps.772 12497556

[31] Zarit SH, Stephens MA, Townsend A, Greene R. Stress reduction for family caregivers: effects of adult day care use. J Gerontol B Psychol Sci Soc Sci. 1998;53:S267-77. 10.1093/geronb/53b.5.s267 9750575

[32] de Jong JD, Boersma F. Dutch psychogeriatric day-care centers: a qualitative study of the needs and wishes of carers. Int Psychogeriatr. 2009;21:268-77. 10.1017/S1041610208008247 19250557

[33] Dröes RM, Meiland FJM, Schmitz MJ, van Tilburg W. Effect of the Meeting Centres Support Program on informal carers of people with dementia: Results from a multi-centre study. Aging Ment Health. 2006;10(2):112-24. 10.1080/13607860500310682 16517486

[34] Droes RM, Breebaart E, Meiland FJ, Van Tilburg W, Mellenbergh GJ. Effect of Meeting Centres Support Program on feelings of competence of family carers and delay of institutionalization of people with dementia. Aging Ment Health. 2004;8:201-11 10.1080/13607860410001669732 15203401

[35] Tretteteig S, Vatne S, Rokstad AMM. The influence of day care centres designed for people with dementia on family caregivers - a qualitative study. BMC Geriatrics (2017) 17(1):5. 10.1186/s12877-016-0403-2 PMC5216603 28056843

[36] Rokstad M, McCabe L, Robertson JM, Strandenæs MG, Tretteteig S, Vatne S. Day care for people with dementia: A qualitative study comparing experiences from Norway and Scotland. Dementia (London). 2019;18(4):1393-409. 10.1177/1471301217712796 28587483

[37] Gústafsdóttir M. The Family's Experience of Sharing the Care of a Person with Dementia with the Services in Specialized Day-Care Units. Dement Geriatr Cogn Disord. 2014;4:344-54 10.1159/000358823 PMC4202610 25337077

[38] Kwok T, Young D, Yip A, F Ho. Effectiveness of day care services for dementia patients and their caregivers Asian J Gerontol Geriatr 2013; 8:9-15.

[39] Phillipson L, Jones SC. Use of day centers for respite by help-seeking caregivers of individuals with dementia. J Gerontol Nurs. 2012;38:24-34. 10.3928/00989134-20120307-05 22420521

[40] Mossello E, Caleri V, Razzi E, Di Bari M, Cantini C, Tonon E, et al. Day Care for older dementia patients: favorable effects on behavioral and psychological symptoms and caregiver stress. Int J Geriatr Psychiatry. 2008;23:1066-72. 10.1002/gps.2034 18481318

[41] Mavall L, Thorslund M. Does day care also provide care for the caregiver? Arch Gerontol Geriatr. 2007;45:137-50. 10.1016/j.archger.2006.10.005 17129621

[42] Zank S, Schacke C. (2002). Evaluation of geriatric day care units: Effects on patients and caregivers. J Gerontol B Psychol Sci Soc Sci. 2002; 57(4):P348-57. 10.1093/geronb/57.4.p348 12084785

[43] Droes RM. In Beweging; over psychosociale hulpverlening aan demente ouderen. [Moving; on psychosocial care for elderly people with dementia.] Nijkerk: Intro.1991.

[44] Finnema E, Drões R, Ribbe M, van Tilburg W. A Review of Psychosocial Models in Psychogeriatrics: Implications for Care and Research. Alzheimer Dis Assoc Disord. 2000;14(2):68-80. 10.1097/00002093-200004000-00004 10850745

[45] Pearlin LI, Mullan JT, Semple SJ, Skaff MM. Caregiving and the stress process: An overview of concepts and their measures. Gerontologist. 1990;30:583-94. 10.1093/geront/30.5.583 2276631

[46] Clare L. Rehabilitation for people living with dementia: A practical framework of positive support. PLoS Med. 2017;14(3):e1002245. 10.1371/journal.pmed.1002245 PMC5340348 28267744

[47] Woods B, Aguirre E, Spector A E, Orrell M. Cognitive stimulation to improve cognitive functioning in people with dementia. Cochrane Database Syst Rev. 2012;(2):CD005562. 10.1002/14651858.CD005562.pub2 22336813

[48] Thuné-Boyle IC, Iliffe S, Cerga-Pashoja A, Lowery D, Warner J. The effect of exercise on behavioral and psychological symptoms of dementia: towards a research agenda. Int Psychogeriatr. 2012;24(7):1046-57. 10.1017/S1041610211002365 22172121

[49] Behrer L. Cognitive plasticity in older adults: effects of cognitive training and physical exercise. Ann N Y Acad Sci. 2015;1337:1-6. 10.1111/nyas.12682 25773610

[50] Clare L, Linden DE, Woods RT, Whitaker R, Evans SJ, Parkinson CH, et al. Goal-oriented cognitive rehabilitation for people with early-stage Alzheimer disease: a single-blind randomized controlled trial of clinical efficacy. Am J Geriatr Psychiatry. 2010;18(10):928-39. 10.1097/JGP.0b013e3181d5792a 20808145

